# Land-use effects on local biodiversity in tropical forests vary between continents

**DOI:** 10.1007/s10531-017-1356-2

**Published:** 2017-05-27

**Authors:** Helen R. P. Phillips, Tim Newbold, Andy Purvis

**Affiliations:** 1 Department of Life Sciences, Imperial College London, Silwood Park Campus, London, SL5 7PY UK; 2 Department of Life Sciences, Natural History Museum, Cromwell Road, London, SW7 5BD UK; 3 Present Address: German Centre for Integrative Biodiversity Research (iDiv) Halle-Jena-Leipzig, Deutscher Platz 5e, 04103 Leipzig, Germany; 4 Department of Genetics, Evolution and Environment, Centre for Biodiversity and Environment Research, University College London, Gower Street, London, WC1E 6BT UK

**Keywords:** Oil palm, Community composition, Species sensitivity, Beta diversity, Extinction filter, Biotic homogenization

## Abstract

Land-use change is one of the greatest threats to biodiversity, especially in the tropics where secondary and plantation forests are expanding while primary forest is declining. Understanding how well these disturbed habitats maintain biodiversity is therefore important—specifically how the maturity of secondary forest and the management intensity of plantation forest affect levels of biodiversity. Previous studies have shown that the biotas of different continents respond differently to land use. Any continental differences in the response could be due to differences in land-use intensity and maturity of secondary vegetation or to differences among species in their sensitivity to disturbances. We tested these hypotheses using an extensive dataset collated from published biodiversity comparisons within four tropical regions—Asia, Africa, Central America and South America—and a wide range of animal and plant taxa. We analysed responses to land use of several aspects of biodiversity—species richness, species composition and endemicity—allowing a more detailed comparison than in previous syntheses. Within each continent, assemblages from secondary vegetation of all successional stages retained species richness comparable to those in primary vegetation, but community composition was distinct, especially in younger secondary vegetation. Plantation forests, particularly the most intensively managed, supported a smaller—and very distinct—set of species from sites in primary vegetation. Responses to land use did vary significantly among continents, with the biggest difference in richness between plantation and primary forests in Asia. Responses of individual taxonomic groups did not differ strongly among continents, giving little indication that species were inherently more sensitive in Asia than elsewhere. We show that oil palm plantations support particularly low species richness, indicating that continental differences in the response of biodiversity to land use are perhaps more likely explained by Asia’s high prevalence of oil palm plantations.

**Electronic supplementary material:**

The online version of this article (doi:10.1007/s10531-017-1356-2) contains supplementary material, which is available to authorized users.

## Introduction

Land-use change is the greatest threat to terrestrial biodiversity in the tropics (Sala et al. 2000; Jetz et al. 2007; Pekin and Pijanowski 2012). Tropical forests are the most biodiverse terrestrial habitat, with around 50% of the world’s species (Dirzo and Raven 2003; Wright 2005), but roughly 68,000 km^2^ of tropical forest is lost annually (FAO and JRC 2012)—an amount that could be increasing by 3% (>2000 km^2^) each year (Hansen et al. 2013). Of the 11 million km^2^ that remain, nearly half (5 million km^2^) is considered to be either degraded (ITTO 2002) or secondary forest that has regrown after human use (e.g. agricultural abandonment and clear felling: Wright and Muller-Landau 2006; Lewis et al. 2015) or natural disturbances (e.g. fires and cyclones: Chazdon et al. 2009). Increasingly, both primary and secondary forests are converted to plantation forest (Koh and Wilcove 2008; Wilcove and Koh 2010; Carlson et al. 2013), especially in Asia where demand for palm oil is a major driver of deforestation (Koh and Wilcove 2007; Fitzherbert et al. 2008). Our aim here was to compare local (site-level) biodiversity among primary, secondary and plantation forests, testing whether differences vary among continents and across a broad set of taxa, and seeking to explain any differences that emerge.

Whether secondary forests are of value for biodiversity conservation has long been of interest. While some studies of particular taxa have reported that secondary vegetation supports high biodiversity (e.g. Barlow et al. 2007a, b; Berry et al. 2010; Struebig et al. 2013), others have not (e.g. Floren and Linsenmair 2005; Bihn et al. 2008; Gibson et al. 2011). One possible source of heterogeneity in effects is that the conservation value of secondary vegetation could increase with successional stage, with older secondary vegetation approaching natural vegetation in terms of structural complexity (DeWalt et al. 2003) and site-level diversity (Brown and Lugo 1990; Veddeler et al. 2005; Martin et al. 2013; Newbold et al. 2015; Norden et al. 2015). In tropical regions, the recovery of site-level diversity can be rapid, sometimes within 20–40 years (Dunn 2004), but assemblages may take much longer than this to approach the species composition seen in primary vegetation (Martin et al. 2013).

Plantation forests tend to have simpler vegetation architecture than primary or secondary forests (Fitzherbert et al. 2008) so it is unsurprising that they support less diverse and compositionally distinct assemblages, across a range of taxa (e.g. vertebrates: Waltert et al. 2004; Sodhi et al. 2005; Freudmann et al. 2015, invertebrates: Nichols et al. 2007; Barlow et al. 2007b; Gardner et al. 2008; Brühl and Eltz 2010; Barnes et al. 2014, mixed: Newbold et al. 2015; 2016a, b). However, not all studies report such differences between natural and plantation forests (e.g. Danielsen et al. 2009), suggesting responses may be heterogeneous. Differences in site-level diversity might be attributable to stand age (Bremer and Farley 2010; Taki et al. 2010; Wang and Foster 2015), or might reflect differences in at least two other factors. First, plantations that are managed less intensively, such as those including shade trees, could retain more of the original biodiversity than more intensive plantations (Faria et al. 2007; Clough et al. 2009; but see la Mora et al. 2013). Such an effect may occur either within a crop type (e.g., cacao plantations with more shade trees often support higher species richness: Clough et al. 2009) or between crop types (e.g. oil palm plantations may be more intensive than other plantations due to their uniform stand age and understorey clearance: Fitzherbert et al. 2008; Foster et al. 2011; Wang and Foster 2015). Second, responses may vary among taxonomic groups (Newbold et al. 2014; Chaudhary et al. 2016): for example, Lawton et al. (1998) found that plantations supported fewer species of bird but more leaf-litter ant species than did primary forest. If any of these factors tend to differ among regions, then the impact seen on biodiversity may also differ regionally, a factor not usually accounted for in large-scale analysis (e.g. Newbold et al. 2015).

Gibson et al. (2011) showed, in a global meta-analysis, that the impact of tropical forest disturbance on biodiversity was more severe in Asia than in other regions (Africa, South America and Central America). There are at least two major reasons why the response of biodiversity to land use might vary among geographic regions, which are not usually accounted for in large-scale analysis (e.g. Newbold et al. 2015). First, variation among regions in the prevalence of different types or intensities of land use or in the sampling of different taxonomic groups, which—as described above—will lead to differences in observed responses of biodiversity to land use. Second, differences in the intrinsic sensitivity of the biota to land-use change or land-use intensity (Gibson et al. 2011; Gerstner et al. 2014; Chaudhary et al. 2016; De Palma et al. 2016; Newbold et al. 2016a). Such differences in sensitivity could arise through regional differences in range size (Orme et al. 2006; Schipper et al. 2008), which probably correlates with ecological flexibility in the face of environmental changes (Bonier et al. 2007; Cardillo et al. 2008; Slatyer et al. 2013), or regional differences in land-use (Achard et al. 2002; Lambin et al. 2003), with longer periods of land use possibly having already filtered out the most sensitive species—a phenomenon referred to as an ‘extinction filter’—meaning that current land-use differences have less of an impact (Balmford 1996). Although biogeography will also play a role in shaping communities within continents (Corlett and Primack 2006), in order to capture these effects data from a greater spatial grain would need to be utilised, which is not available for this study.

Extinction filters, and disturbance generally, can affect more than merely numbers of species. By favouring the establishment of ecologically-flexible or disturbance-tolerant species, at the expense of disturbance-intolerant endemics, they can cause biotic homogenization (McKinney and Lockwood 1999; Arroyo-Rodríguez et al. 2013; Püttker et al. 2015; de Solar et al. 2015), i.e., an increase in similarity between communities in different places. We assess biotic homogenisation in two ways: first, by analysing compositional turnover (beta diversity) between pairs of sites (McKinney 2006; Devictor et al. 2008; Karp et al. 2012); and second, by using the distribution of species’ range sizes at a site, with more disturbed sites predicted to be more dominated by wide-ranging species than more natural sites (Mandle and Ticktin 2013).

We compared effects of land use on local biodiversity across four tropical regions—Asia, Africa, Central America and South America—and across a broad range of taxa. Because of the need for geographic and taxonomic breadth, we used data from the PREDICTS database (Hudson et al. 2014, 2016), a large compilation of data from published spatial comparisons of ecological assemblages at sites facing different anthropogenic pressures. We used a range of measures of biodiversity to capture effects on beta as well as alpha diversity, focusing on three main questions; (1) How do secondary vegetation age and plantation intensity mediate the response of biodiversity to land use change? (2) Does the effect of land use on local biodiversity vary among continents in the tropics? (3) Are any among-continent differences more consistent with differences in the intensity of human land use or with differences in the sensitivity of the biota?

## Methods

**Fig. 1:**
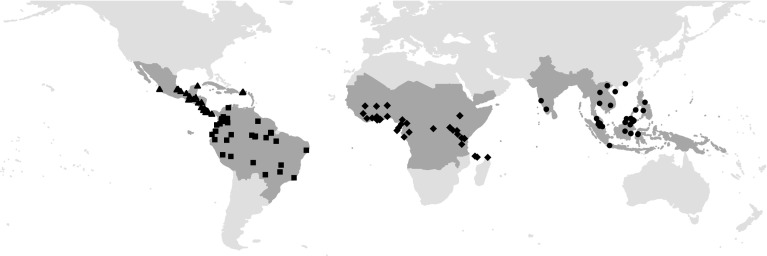
Location of the 144 studies from which data were acquired, with the 35 countries represented grouped into four continents: Central America (*triangles*), South America (*squares*), Africa (*diamonds*) and Asia (*circles*)

The PREDICTS database, described in full by (Hudson et al. 2014, 2016), is a large—but inevitably far from comprehensive—collation of data from published studies worldwide that have compared biodiversity (typically the abundances or occurrences of sets of species, but sometimes simply species richness) of community assemblages at multiple sites differing in the nature and/or intensity of the human pressure faced. Data used here were contributed to the PREDICTS project by many researchers and collated into the database by the project team between March 2012 and December 2015 following many structured and opportunistic literature searches (see Supplementary Material Appendix A and Supplementary Fig. S1 for publication bias analysis). 140 articles collated in the PREDICTS database were suitable for this analysis (Fig. 1). Each article that provided data was collated as a ‘source’. When a source separated data collected using different methodologies (for example, if multiple taxonomic groups were sampled using different techniques), it was split into corresponding ‘studies’, within which site-level diversity estimates are comparable. A study contained a set of sites, with each site comprising a single or multiple sampled plots (i.e. a quadrat or transect, see Hudson et al. 2014 for details).

Using information in the source papers or provided by those papers’ authors, sites were categorised into eight land-use types: primary vegetation, mature secondary vegetation, intermediate secondary vegetation, young secondary vegetation, plantation forest, pasture, cropland and urban (see Hudson et al. 2014 for detailed descriptions of land use categories). In this study, we focused on sites in primary vegetation, secondary vegetation and plantation forest (Table 1). Primary vegetation included sites where there had been some disturbance, e.g. selective logging, but not complete removal or considerable destruction of vegetation. The PREDICTS database also contains categorical information on the intensity of disturbance at each site, which could be important if responses within primary forest vary with disturbance level (Barlow et al. 2016). We chose to not include disturbance levels within primary forest due to the lack of data across the all disturbance levels for the four continent. However, we have shown previously (Newbold et al. 2015) that disturbance within primary vegetation has minimal impact on average numbers of species and individuals sampled at sites. Although previous studies have investigated age of secondary vegetation with a continuous variable (e.g. Dunn 2004), the PREDICTS database collates secondary vegetation into categories based on successional stage rather than age to allow comparison between different biomes and to ensure that the maximum number of studies can be included. In addition, for some of our analyses (specifically the “focal-taxon models” discussed below), to ensure adequate sample sizes for modelling, we combined primary vegetation and secondary vegetation (encompassing all stages of recovery) into a single class, “Natural”.

**Table 1:** Definitions of land use categories based on site level characteristics, showing numbers of studies and sites in the dataset used in this investigation

Predominant Habitat	Land use category	Description	Studies	Sites
Primary vegetation	Primary vegetation	No evidence of prior complete or near complete destruction of the natural vegetation	150	2453
Secondary vegetation	Mature secondary vegetation	Regeneration following complete removal of primary vegetation; architectural structure approaching that of primary vegetation, corresponding to a completed succession	32	172
	Intermediate secondary vegetation	Regeneration following complete removal of primary vegetation; mixed architecture showing a mid-successional stage	56	466
	Young secondary vegetation	Regeneration following complete removal of primary vegetation; simple architecture representing an early successional stage	46	334
Plantation forest	Low-intensity plantation forest	Often have understorey growth, as well as shade trees with minimal amount of pesticides and fertilizers	47	419
	Medium-intensity plantation forest	Monocultures, but with limited amount of pesticides and fertilizers	47	970
	High-intensity plantation forest	Monocultures with extensive use of pesticides and fertilizers, and usually regular clear felling	22	87

See Hudson et al. (2014) for more detailed information on all land use classification

We used only those sites located within the tropics (latitude < ±23°). These data were split into four continents—Asia, Africa, South America and Central America—following Gibson et al. (2011), despite Central and South America’s geographical proximity and similar tree communities (Slik et al. 2015). Tropical Australasia and Melanesia provided too few sites for modelling so were excluded from the analysis.

We excluded studies that did not have sites from at least two of the focal land uses. Plantation-forest sites were classified into three management-intensity classes (low, medium and high), resulting in seven land use classes defined in Table 1 (using the same definitions as Hudson et al. 2014). The plantation crop was classified into one of the following six categories: wood, fruit/vegetables, coffee, cocoa, oil palm, and a local mixture of crops. Although it has been shown that the response of biodiversity can depend upon the timber systems and management practice (Bicknell et al. 2014; Burivalova et al. 2014; Chaudhary et al. 2016), our dataset contained too few sites within the “wood” category to further categorise based on intensity or product. Expansion of rubber plantations is also considered to be another strong driver of land use change and therefore biodiversity loss within the tropics (Ziegler et al. 2009). There were too few rubber-plantation sites to model them separately, so they were therefore grouped in the “wood” category. Studies where the sampling focused on a single species or a predetermined list of species (rather than recording any species within the focal taxonomic or ecological group that was sampled) were removed to avoid biasing species-richness estimates. The taxonomic focus of each study was coarsely classified into three higher taxa: vertebrates, invertebrates and plants (Table 2). Finer divisions (e.g., arthropod orders) would have reduced sample sizes too far to permit robust modelling. Too few data were available for fungi to permit modelling so these were excluded.

**Table 2:** Numbers of sites in the dataset within each Higher Taxon category and the broad taxonomic groups that they contain for each continent

Higher taxon	Asia	Africa	Central America	South America
Vertebrates	243	1130	401	331
Amphibia	10 (1)	61 (2)	129 (4)	27 (3)
Aves	143 (7)	796 (12)	89 (5)	218 (10)
Mammalia	79 (7)	264 (6)	10 (2)	83 (7)
Reptilia	11 (2)	9 (1)	173 (4)	3 (1)
Invertebrates	462	239	160	591
Mollusca	0	36 (1)	0	0
Arachnida	8 (1)	9 (1)	6 (1)	237 (4)
Blattodea	0	8 (1)	0	0
Coleoptera	290 (4)	37 (3)	24 (2)	65 (6)
Diptera	0	36 (1)	0	6 (2)
Hymenoptera	116 (9)	5 (1)	69 (6)	262 (12)
Lepidoptera	48 (9)	108 (6)	58 (2)	18 (2)
Odonata	0	0	3 (1)	0
Orthoptera	0	0	0	3 (1)
Plants	334	821	169	20
Bryophyta	0	84 (2)	0	4 (1)
Pinopsida	0	0	60 (1)	0
Polypodiopsida	0	63 (2)	0	4 (1)
Liliopsida	0	0	13 (1)	0
Magnoliopsida	334 (10)	652 (9)	96 (4)	12 (2)

Numbers in brackets are the numbers of studies that sampled each taxonomic group (some studies sampled multiple groups)

Several diversity measures were calculated at each site to use as response variables in the models. Within-sample species richness (hereafter, species richness) was the number of species sampled at a site. For sites with abundance data, Simpson’s evenness was calculated by dividing the inverse of Simpson’s D (Smith and Wilson 1996) by the site’s species richness. Community weighted mean (CWM) log_e_ range size was calculated as a simple measure of biotic homogenisation (Mandle and Ticktin 2013) as follows. For every taxon identified to species level by a common or scientific name (see Hudson et al. 2014 for more information on taxonomic identity), the species’ range size was estimated as the sum of the areas of 1° grid cells containing a Global Biodiversity Information Facility (GBIF) record (queried on 11th February 2014). These range estimates were log_e_-transformed to reduce skew. For each site with species abundance data, CWM log_e_ range size was calculated as a weighted mean of the log-transformed species’ values, where the weights were species’ abundances. The mean log_e_ range size was calculated for sites where no abundance data was available. This approach has previously been shown to correlate with abundance weighted mean, for characteristics of species other than range size, without biasing results (Newbold et al. 2012).

Site-level species richness, Simpson’s evenness and CWM log_e_ range size were used as response variables in linear mixed-effects models, which allow for nested and heterogeneous data (Bolker et al. 2009; Zuur et al. 2009). Species-richness models used Poisson errors with an observation-level random effect (Harrison 2014) to account for overdispersion (tested using the sum of the squared Pearson residuals and the ratio of the residual degrees of freedom), models of Simpson’s evenness and CWM log_e_ range size used Gaussian errors. We first modelled each response variable in turn with continent, higher taxonomic group, the full land-use classification (Table 1) and all two-way interactions as fixed effects. We refer to these models as “land-uses models”. To test whether the sensitivity of individual taxa varied among continents to land use (i.e., to exclude confounding effects of spatial biases in the taxa sampled), four additional species richness models were used on different taxonomic subsets of the dataset: Aves, herptiles (reptiles and amphibians), Lepidoptera and Hymenoptera (henceforth called “focal-taxon models”). The fixed effects in the focal-taxon models were continent and land use (natural versus non-natural), and the interaction between them. Each focal-taxon model was fitted separately (rather than include taxon as an interacting fixed effect) because there were insufficient data for some taxonomic groups to model natural versus non-natural in each continent. To test whether differences among continents could have been caused by differences in plantation crop, species richness was further modelled with all land uses, but with plantation sites split by crop type, and higher taxon (vertebrate vs invertebrate; plant data were too sparse) as an interacting fixed effect (“Crop-type models”).

Each model’s fixed effects were simplified using backwards stepwise selection based on log-likelihood values (Murtaugh 2009). Interacting variables were removed first if P > 0.05, followed by any single variables that were not involved in remaining interactions and where P > 0.05 (Zuur et al. 2009). The random-intercepts structure for all models accounted for variation among sources and studies—for example, differences in methodology and location—and, within studies, the spatial structure of sites in the experimental design (i.e., spatial blocks of sites). Random slopes were also considered but their use was found to be unfeasible owing to the sample size.

In addition to the site-level measures of diversity, we estimated the level of biotic homogenization between each site’s community and the average community in primary forest. This was calculated using Sørensen’s index (Magurran 2004), which quantifies the dissimilarity in species composition between two communities based on the number of shared species. Within each study with at least one primary vegetation site, the Sørensen’s index was calculated for all pairwise comparisons between sites, and the average calculated for each land-use pair (e.g., primary vegetation vs secondary vegetation). The resulting similarities were rescaled within each study so that average similarity of pairs of sites in primary vegetation sites was 1 (Newbold et al. 2015). These rescaled similarities were then averaged across studies within each continent separately, to create continent-specific matrices of compositional similarity between each pair of land uses. A similar analysis was performed focusing on the plantation land-use category, splitting the sites by crop type, to produce a single, global matrix. Matrices were then visualized as dendrograms, created from the inverse of the pairwise dissimilarity matrix using the R function hclust, using the “complete linkage” method, which clusters based on similarity.

All analyses were conducted in R (version 3.2.1, R Core Team 2015) using mixed-effects models in the lme4 package (version 1.1–9, Bates et al. 2015); P values were obtained using the ‘Anova’ function in the ‘car’ library (version 2.1–0, Fox and Weisberg 2011).

## Results

In total, 184 studies in the PREDICTS database, from 140 published articles (See Supplementary Material Appendix B for publication list), met the criteria for inclusion in this study, representing over 12,000 species. The 4901 sites were located within 39 countries (Fig. 1), 3413 within the Tropical and Subtropical Moist Broadleaf Forest biome (Supplementary Table S1; The Nature Conservancy 2009), but land uses were reasonably equally distributed across continents (Variance Inflation Factor <3, Zuur et al. 2010; analysis not presented, but see Table 3). Sites were sampled between 1992 and 2011 (median = 2006). Simpson’s evenness (which required abundance, not occurrence data) and CWM log_e_ range size (which required GBIF records for matching species) could be calculated for 4029 and 4332 of the sites respectively, and Sørensen’s index for 130 of the 184 studies.

**Table 3:** The total number of sites (N_sites_) and the total number of studies (N_studies_) in each land use within each continent and overall

Land use	Asia	Africa	Central America	South America	All
Primary vegetation	N_sites_ = 528	N_sites_ = 1079	N_sites_ = 193	N_sites_ = 653	N_sites_ = 2453
	N_studies_ = 45	N_studies_ = 43	N_studies_ = 22	N_studies_ = 40	N_studies_ = 150
Mature S.V	N_sites_ = 16	N_sites_ = 73	N_sites_ = 66	N_sites_ = 17	N_sites_ = 172
	N_studies_ = 6	N_studies_ = 11	N_studies_ = 7	N_studies_ = 9	N_studies_ = 32
Intermediate S.V	N_sites_ = 61	N_sites_ = 145	N_sites_ = 148	N_sites_ = 112	N_sites_ = 466
	N_studies_ = 9	N_studies_ = 10	N_studies_ = 12	N_studies_ = 25	N_studies_ = 56
Young S.V	N_sites_ = 87	N_sites_ = 124	N_sites_ = 58	N_sites_ = 65	N_sites_ = 334
	N_studies_ = 11	N_studies_ = 14	N_studies_ = 9	N_studies_ = 12	N_studies_ = 46
Low-intensity Plantation Forest	Cocoa (53.7%)	Mixture (44.4%)	Cocoa (55.1%)	Wood (55.6%)	Mixture (27%)
	N_sites_ = 82	N_sites_ = 232	N_sites_ = 69	N_sites_ = 36	N_sites_ = 419
	N_studies_ = 12	N_studies_ = 13	N_studies_ = 6	N_studies_ = 16	N_studies_ = 47
Medium-intensity Plantation Forest	Oil palm (78.4%)	Mixture (60.5%)	Cocoa (89.3%)	Coffee (59.5%)	Mixture (32.8%)
	N_sites_ = 241	N_sites_ = 519	N_sites_ = 168	N_sites_ = 42	N_sites_ = 970
	N_studies_ = 16	N_studies_ = 13	N_studies_ = 8	N_studies_ = 10	N_studies_ = 47
High-intensity Plantation Forest	Oil palm (50%)	Oil palm (99.4%)	Coffee (64.3%)	Coffee (70.6%)	Coffee (34.5%) Oil palm (33.3%)
	N_sites_ = 24	N_sites_ = 18	N_sites_ = 28	N_sites_ = 17	N_sites_ = 87
	N_studies_ = 9	N_studies_ 4	N_studies_ = 4	N_studies_ = 5	N_studies_ = 22

For plantation forests, we also state the most abundant plantation crop type within our sample of sites, in each continent and overall, within each plantation forest intensity category. Secondary vegetation has been abbreviated to S.V

The predominant plantation crop for each intensity category varied among continents (Table 3), with representation of different crops within the PREDICTS database being very uneven among continents (although the Variance Inflation Factor <3, Zuur et al. 2010; analysis not presented). This unevenness may partly reflect geographic patterns of different crops but certainly also reflects current limitations of the database: for example, oil palm was sampled only in Asia and Africa, despite also being prevalent in South America, and coffee plantations were not sampled in Asia, although they are present there.

**Fig. 2:**
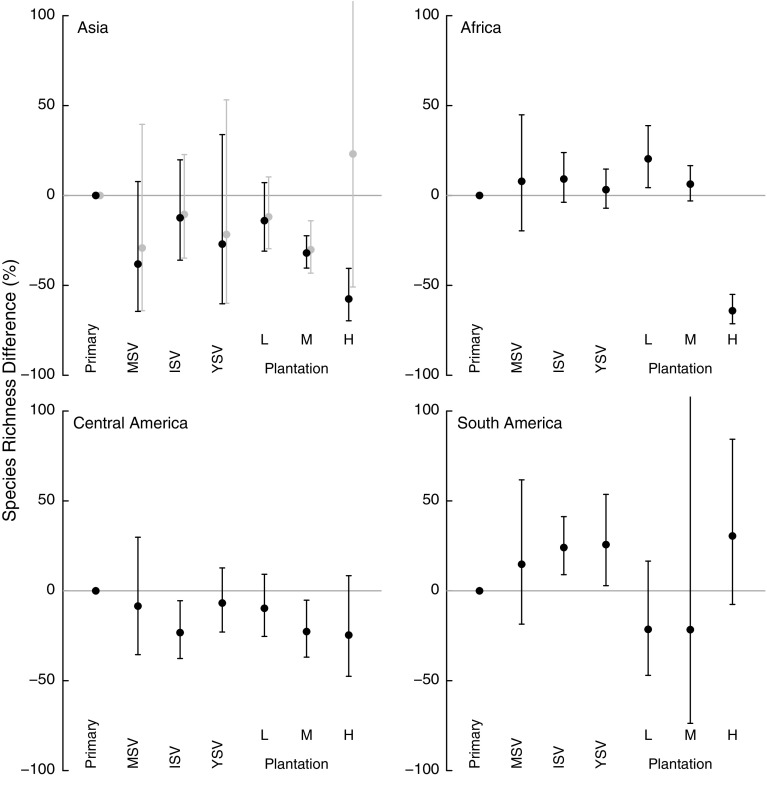
Effects of seven land uses on site-level species richness for Asia, Africa, Central America and South America. From* left* to* right*, land uses are: primary vegetation (‘Primary’); mature secondary vegetation (‘MSV’); intermediate secondary vegetation (‘ISV’); young secondary vegetation (‘YSV’); low-intensity (‘L’), medium-intensity (‘M’) and high-intensity (‘H’) plantation forests (‘Plantation’). Primary vegetation is used as the reference level, and changes in diversity in other land uses is measured relative to this baseline.* Error bars* show 95% CIs. *Grey points* show post hoc analysis of impact on species richness of land use, using only sites in Asia but excluding oil-palm plantation sites

The land-uses model for species-richness was simplified with the removal of one interaction (continent x higher taxon; Table 4). Species richness often did not differ significantly between primary and secondary vegetation within a continent; when it did, sites in intermediate and young secondary vegetation tended to have more species than primary vegetation. Richness in plantation forests—especially high-intensity ones—was usually significantly lower than in primary vegetation; the size of this effect varied among continents, being biggest in Asia and Africa (Fig. 2).

**Table 4:** χ^2^ values for all remaining terms within the mixed effects models

Model		Land use (6)	Taxon (2)	Continent (3)	Land use: continent (18)	Land use: taxon (12)	Continent: taxon (6)
Land-uses models							
Species richness		521.05*	7.69*	2.69*	184.04*	245.58*	–
Simpson’s evenness		38.09*	18.50*	7.24	64.37*	64.60*	–
CWM log_e_ range size		581.05*	94.47*	11.24*	227.81*	229.70*	20.20*

Model			Continent (3)		Natural/non-natural (1)	Natural/non-natural: continent (3)	
Focal-taxon models							
Aves			9.69*		39.69*	38.11*	
Herptiles			4.13		4.24*	15.60*	
Hymenoptera			0.65_d.f.=2_		6.36*	11.73*_d.f.=2_	
Lepidoptera			3.91_d.f.=2_		2.64	23.15*_d.f.=2_	

Model			Taxon (1)		Crop type (9)	Crop type: taxon (9)	
Crop type			0.04		305.67 *	64.99 *	

* P < 0.05

*Dash* indicates that the term was removed upon simplification. Degrees of freedom of terms are shown in brackets next to the model term. In addition, the ‘Continent’ term had reduced degrees of freedom in some of the ‘Focal-Taxon’ models, owing to some taxa not being represented in all continents

**Fig. 3:**
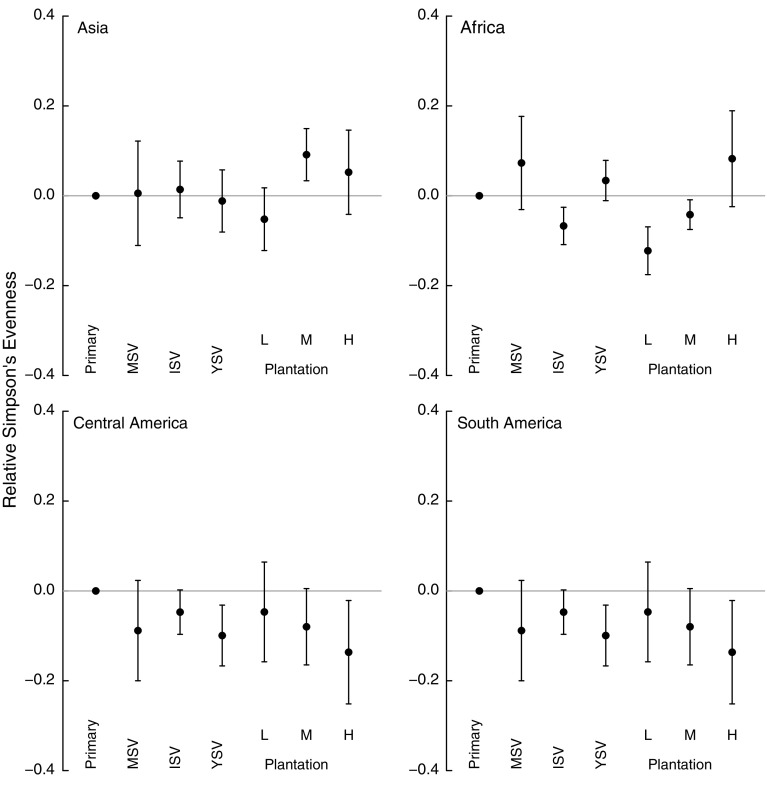
Effects of seven land uses on site-level Simpson’s evenness for Asia, Africa, Central America and South America. Land uses are as in Fig. 2. *Error bars* show 95% CIs

The continent × higher taxon interaction was also removed from the land-uses model for Simpson’s evenness during model simplification (Table 4). The response of Simpson’s evenness to land use pressures differed among continents (Fig. 3), with secondary vegetation and plantation forest typically having substantially lower values than primary vegetation in Africa, Central and South America but not in Asia.

**Fig. 4:**
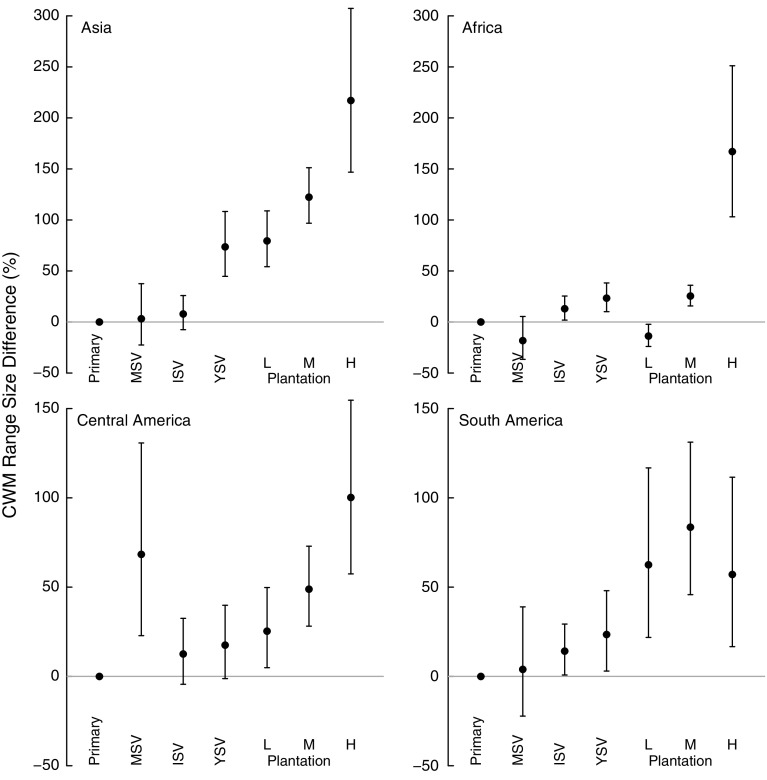
Effect of land use on community weighted mean (CWM) range size for Asia, Africa, Central America and South America. Land uses are as in Fig. 2. *Error bars* show 95% CIs

All interaction terms had a significant effect on the land-uses model for CWM log_e_ range size (Table 4). Within continents, mature secondary vegetation usually had a similar CWM log_e_ range size to primary vegetation, but young and intermediate secondary vegetation sites had higher average range sizes than primary vegetation in all continents (Fig. 4). Plantation forest was usually associated with significantly larger CWM log_e_ range size than primary vegetation. High-intensity plantations in Asia showed the largest increase, with an absolute CWM range nearly 2.5 times larger than that found in primary vegetation (Fig. 4). In Africa, high-intensity plantation was the only land use showing a large increase in CWM log_e_ range (Fig. 4), but the paucity of GBIF records for African species (Supplementary Fig. S2) provides grounds for caution about this result.

**Fig. 5:**
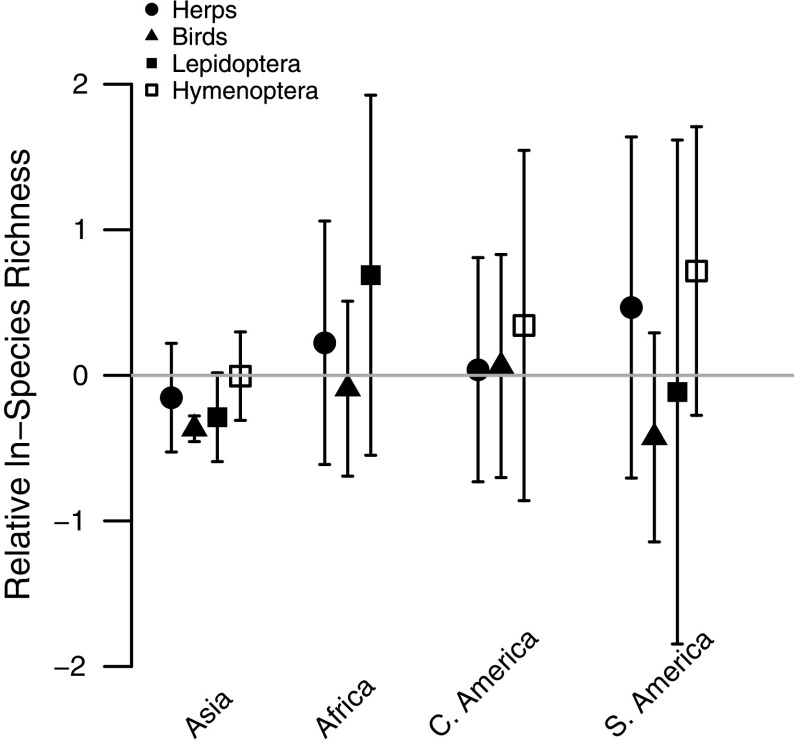
Effects of non-natural land uses (plantation forest) on site-level ln-species richness in each of four taxa (*circles* herptiles, *triangles* Aves, *filled square* Lepidoptera, *empty square* Hymenoptera) for Asia, Africa, Central America and South America, relative to the baseline of site-level ln-species richness in natural land uses (primary vegetation, secondary vegetation), indicated by the zero-line. *Error bars* show 95% CIs

All focal-taxon models maintained the interaction between continent and natural versus non-natural, but there was no indication that any taxon’s response to non-natural land use differed strongly among continents (Table 4; Fig. 5).

**Fig. 6:**
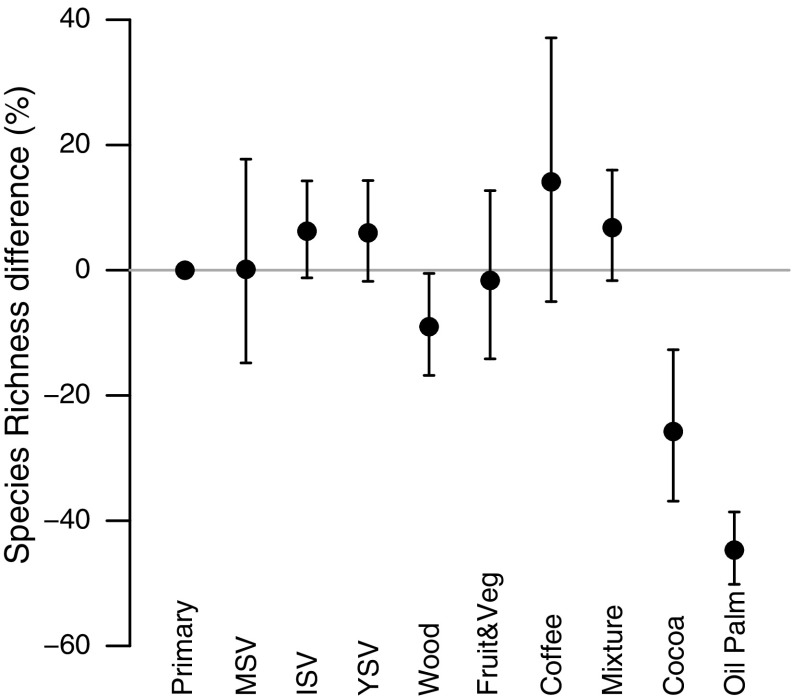
The average site-level species richness within each plantation crop type. Abbreviations as in Fig. 2, with the addition of the following plantation crop categories; Coffee, Oil Palm, Cocoa, Wood, Mixture (a local mixture of crops) and Fruit & Veg (fruit or vegetable crops). *Error bars* show 95% CIs

When modelling effects of plantation-crop type as well as land use on species richness, the interaction between higher taxon and land use was retained during model simplification (Table 4), but is not considered further as not all higher taxon were represented in all plantation-crop types. Oil palm supported fewest species and coffee the most (Fig. 6).

**Fig. 7:**
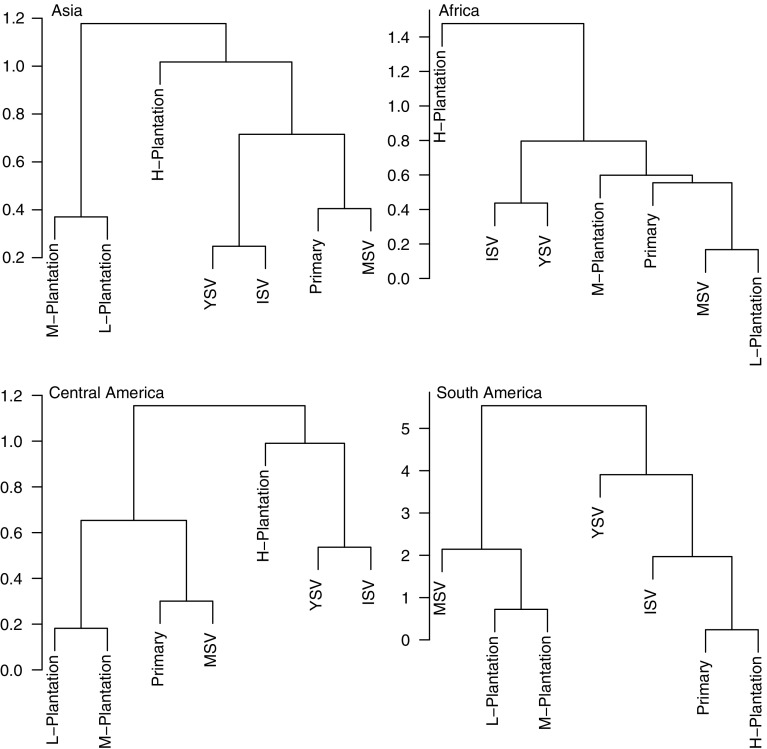
Dendrogram of the average community compositional similarity (based on Sørensen’s similarity index) of each land use compared to every other, within each continent; Asia, Africa, Central America and South America. *Primary* primary vegetation, *MSV* mature secondary vegetation, *ISV* intermediate secondary vegetation, *YSV* young secondary vegetation, *L-plantation* low-intensity plantation forest, *M-plantation* medium-intensity plantation forest, and *H-plantation* high-intensity plantation forest

**Fig. 8:**
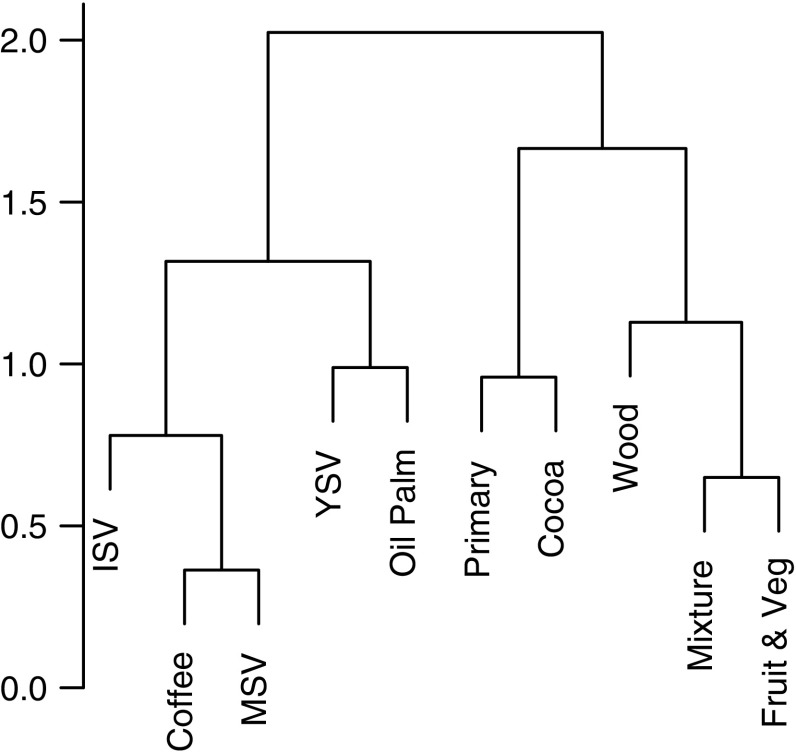
Dendrogram of the average community similarity (based on Sørensen’s similarity index) of plantation crop types and the other non-plantation land uses across all continents. Abbreviations as in Fig. 7, with the addition of the following plantation crop categories; Coffee, Oil Palm, Cocoa, Wood, Mixture (a local mixture of crops) and Fruit & Veg (fruit or vegetable crops)

Compositional similarity between land uses showed considerable variation among continents (Fig. 7). Different management intensities of plantation forest tended to cluster together or with the secondary vegetation categories, rather than with the primary vegetation. When community composition was compared among plantation crop types, across all continents, secondary vegetation was grouped together with coffee and oil palm (with oil palm having communities more like young secondary vegetation, and coffee having communities more like intermediate or mature secondary vegetation), forming a distinct cluster from primary vegetation and the remaining crop types (Fig. 8).

## Discussion

For all measures that we analysed, the response of site-level biodiversity to land use differed significantly among the four continents. Such differences might be expected given that continents also differ in biophysical, evolutionary and socio-economic history (Sodhi et al. 2004; Corlett and Primack 2006; Gardner et al. 2009, 2010). These previous studies, as well as the results of Gibson et al. (2011), suggest that global or pan-tropical studies should consider continental differences. However, when we fitted models to particular higher taxa, there was no consistent tendency for the effect of human land use on species richness to be more severe in any one continent, suggesting that any continental differences in the inherent sensitivity of the biodiversity are not general across these taxa. There was an indication that some land uses, particularly more intensive plantation forests, have larger impacts on biodiversity than others, and the effect of these land uses might be more pronounced in Asia.

Plantations generally supported fewer, more widely-distributed species than either primary or secondary vegetation (Figs. 3, 4). The magnitude of the effects varied among continents and with land-use intensity: intensively used plantations in Asia and Africa had particularly low species richness. These results agree with previous studies that found plantations to be highly detrimental to biodiversity (Barlow et al. 2007b; Brühl and Eltz 2010; Edwards et al. 2010; Freudmann et al. 2015; Gilroy et al. 2015), especially if they are managed intensively (Faria et al. 2007; Clough et al. 2009; Tadesse et al. 2014; Newbold et al. 2015). The low biodiversity in intensive plantations is likely to reflect the lack of structural complexity and the homogeneity in the age of the stands (Fitzherbert et al. 2008; Clough et al. 2009; Foster et al. 2011; Freudmann et al. 2015). In Asia and Africa, the most common crop in the high-intensity plantations was oil palm, which supports fewer species than the other plantation crops in our study (Fig. 6). Indeed, if oil palm data are from removed from the Asian sites, the high-intensity plantations do not show a significant difference in species richness compared with primary vegetation (analysis not shown, but results shown in Fig. 2 in grey). Considering how widespread oil palm already is in the tropics (Koh and Wilcove 2007; Wilcove and Koh 2010; Carlson et al. 2013), and its rapid ongoing expansion of 9% per year (Fitzherbert et al. 2008), its effects on biodiversity are particularly concerning for conservation.

Central and South American plantations impacted species richness less than those in Asia and Africa. This may reflect the prevalence of coffee crops in the high-intensity plantations in our data set for Central and South America. Our data were insufficient to model management intensity alongside crop identity, meaning we could not test this possibility. However, most of the Neotropical high-intensity coffee plantations in our dataset had shade trees (even though these were usually of just a single species), perhaps providing more structural complexity than in high-intensity plantations elsewhere (Tadesse et al. 2014).

Many previous studies have shown lower species richness in secondary than primary vegetation (Barlow et al. 2007a; Gibson et al. 2011; Klimes et al. 2012), especially younger secondary vegetation (DeWalt et al. 2003; Veddeler et al. 2005; Bihn et al. 2008; Norden et al. 2009; Newbold et al. 2015), perhaps because the vegetation lacks the complexity needed to maintain high levels of biodiversity. Although we found that assemblages in primary and secondary vegetation did not differ strongly in species richness, the differences in average range size and Simpson’s evenness highlight that the similarity in species richness hides differences in abundance and species identity—sites in secondary vegetation have gained some species, particularly wide-ranged species, but lost others, particularly narrow-ranged species (Struebig et al. 2013; McGill et al. 2015). This illustrates a more general pattern: land-use change is not only causing a loss of species but also a shift in community composition (de Solar et al. 2016; Newbold et al. 2016b) towards more widespread species, resulting in biotic homogenisation (McKinney and Lockwood 1999; McKinney 2006; Ranganathan et al. 2008; Karp et al. 2012; Mandle and Ticktin 2013; McGill et al. 2015; de Solar et al. 2015).

Caution is needed in interpreting our results about average range sizes, owing to collection biases in the records held by GBIF (Yesson et al. 2007; Newbold 2010; Meyer et al. 2015). In particular, our results for Africa, and for some land-uses in Central and South America, should be taken as preliminary because many of the sites from those regions had low coverage of species in GBIF (Supplementary Fig. S2). Additionally, our use of large grid cells limits the precision of range-size estimates, especially for small-ranged species. However, the worst of the biases in GBIF records are between taxa and large regions (Meyer et al. 2015), rather than within them; our use of hierarchical mixed-effects models, and the fact that most of our studies are taxonomically fairly restricted (Hudson et al. 2014), means that we do not typically make direct comparisons across taxa and regions. For the vertebrates within the PREDICTS database, there is positive correlation between the mean range size estimates based on GBIF data and IUCN range maps (R^2^ = 0.63, analysis not presented); however, as our knowledge of species distributions improves, future studies could incorporate more accurate range estimates for species, which should improve precision and make interpretation easier.

This study used spatial comparisons of compositional similarity between pairs of sites, which cannot provide complete evidence of biotic homogenisation because they do not directly consider temporal changes (Olden and Rooney 2006). Many previous studies of biotic homogenisation have analysed changes over time (e.g. Olden and Rooney 2006; Lôbo et al. 2011); however, data on spatially distinct communities are much more widely available than temporal data (McKinney 2006) and have increasingly been used to quantify homogenization (Baiser et al. 2012; de Solar et al. 2015). If anything, spatial comparisons are likely to underestimate the effects of land conversion on biodiversity (França et al. 2016).

## Conclusions

Overall, our results suggest that the response of biodiversity to land use varies markedly among continents, but that this heterogeneity is more likely to reflect differences in the intensity of land-use pressures experienced, or combined taxonomic and spatial biases in sampling, rather than systematic differences in the intrinsic sensitivity of species among regions. Although some trends were consistent among continents, our study highlights benefits of accounting for continental differences in pan-tropical analyses, to account for variation in the prevalence of different crop types and for biases in sampling (for example, of different taxonomic groups). Overall, our results suggest that to reduce species loss and retain species composition, the intensity of plantations forests should be reduced, either a reduction in management intensity or the crop grown. Considering that oil palm, the most detrimental plantation for biodiversity in our study, is still expanding across the tropics, especially in the Americas, the implication of these results is timely. Although assemblages in mature secondary vegetation approach those in primary vegetation in terms of species richness, they tend to be compositionally very distinct, emphasizing the irreplaceability of primary forests (Gibson et al. 2011) and the limitations of species richness as a biodiversity metric (Dornelas et al. 2014). The maintenance and expansion of forests globally provide one route to climate change mitigation (Hurtt et al. 2011); however, although primary, secondary and plantation forests may provide similar services in terms of carbon capture (Martin et al. 2013; Poorter et al. 2016), they support profoundly different ecological assemblages.

## Electronic supplementary material

Below is the link to the electronic supplementary material.

Supplementary material 1 (DOCX 514 kb)
