# Supplementary material for: Land-use effects on local biodiversity in tropical forests vary between continents
Source: Biodivers Conserv. 2017 May 27;26(9):2251–70. doi: 10.1007/s10531-017-1356-2 (PMC6979682; doi:10.1007/s10531-017-1356-2)
Supplement: Supplementary file 1 — Supplementary material 1 (DOCX 514 kb) [file 10531_2017_1356_MOESM1_ESM.docx]

**Land-use effects on local biodiversity in tropical forests vary between continents
Submitted to: Biodiversity and Conservation**

Helen R.P. Phillips

Department of Life Sciences, Imperial College London, Silwood Park Campus, London, SL5 7PY

Department of Life Sciences, Natural History, Cromwell Road, London SW7 5BD

Current Address: German Centre for Integrative Biodiversity Research (iDiv) Halle-Jena-Leipzig, Deutscher Platz 5e, 04103 Leipzig, Germany

Email: helen.phillips@idiv.de

Tim Newbold

and

Andy Purvis

**Supplementary Material: Appendix A**

It is expected that meta-analysis (calculation of effect sizes from primary literature) and synthetic analysis (re-analysis of raw data from a collection of publications) might suffer from publication bias. In other words, significant results are more likely to have been published than non-significant results. In meta-analysis multiple methods exist to test for and correct publication bias, such as the construction of funnel plots to visualise the relationship between effect size and a measure of variance (Koricheva et al. 2013). Newbold et al. (2015) adapted this method to test for publication bias when modelling raw site-level diversity values in mixed-effects models (i.e. when no per-study effect sizes are utilised). When variance is high, effect sizes should be highly variable and spread both sides of the overall effect size. When variance is low, effect sizes should be clustered around the overall effect size. We followed the methods of Newbold et al. (2015) to test for publication bias within this dataset.

For each study within the dataset which contained at least two land uses with at least two sites in each, a generalised linear model was fitted relating species richness to land use. The estimated coefficient (effect size) for each land use sampled (relative to the reference level) was plotted against its standard error. These study level coefficients were then compared to the coefficients taken from the species richness model used in the main analysis. For most of the land uses there was some indication of publication bias, with effect sizes with the largest standard error predominantly having highly negative effect sizes. However, in most cases, these effect sizes came from studies with relatively few sites, and thus contribute little weight to the model. Removing studies where any land use had less than 3 sites, and re-analysing the land-use model with species richness as a response variable did not significantly change the outcomes (analysis not presented). All the studies with larger numbers of sites had relatively small standard errors.

**References**

Koricheva J, Gurevitch J, Mengersen K (2013) Handbook of meta-analysis in ecology and evolution.

Newbold T, Hudson LN, Hill SLL, et al (2015) Global effects of land use on local terrestrial biodiversity. Nature 520:45–50.

**Supplementary Material: Figure S1**

**Figure S1:** For each study, the estimated coefficient (effect size) for each land use sampled relative to the reference level was plotted against its standard error. Dotted horizontal lines show the coefficients taken from the species richness model used in the main analysis. Size of the point indicates the number of sites of the land use being compared. L-Plantation forest = low-intensity plantation forest, M-Plantation forest = medium-intensity plantation forest, and H-Plantation forest = high-intensity plantation forest

**
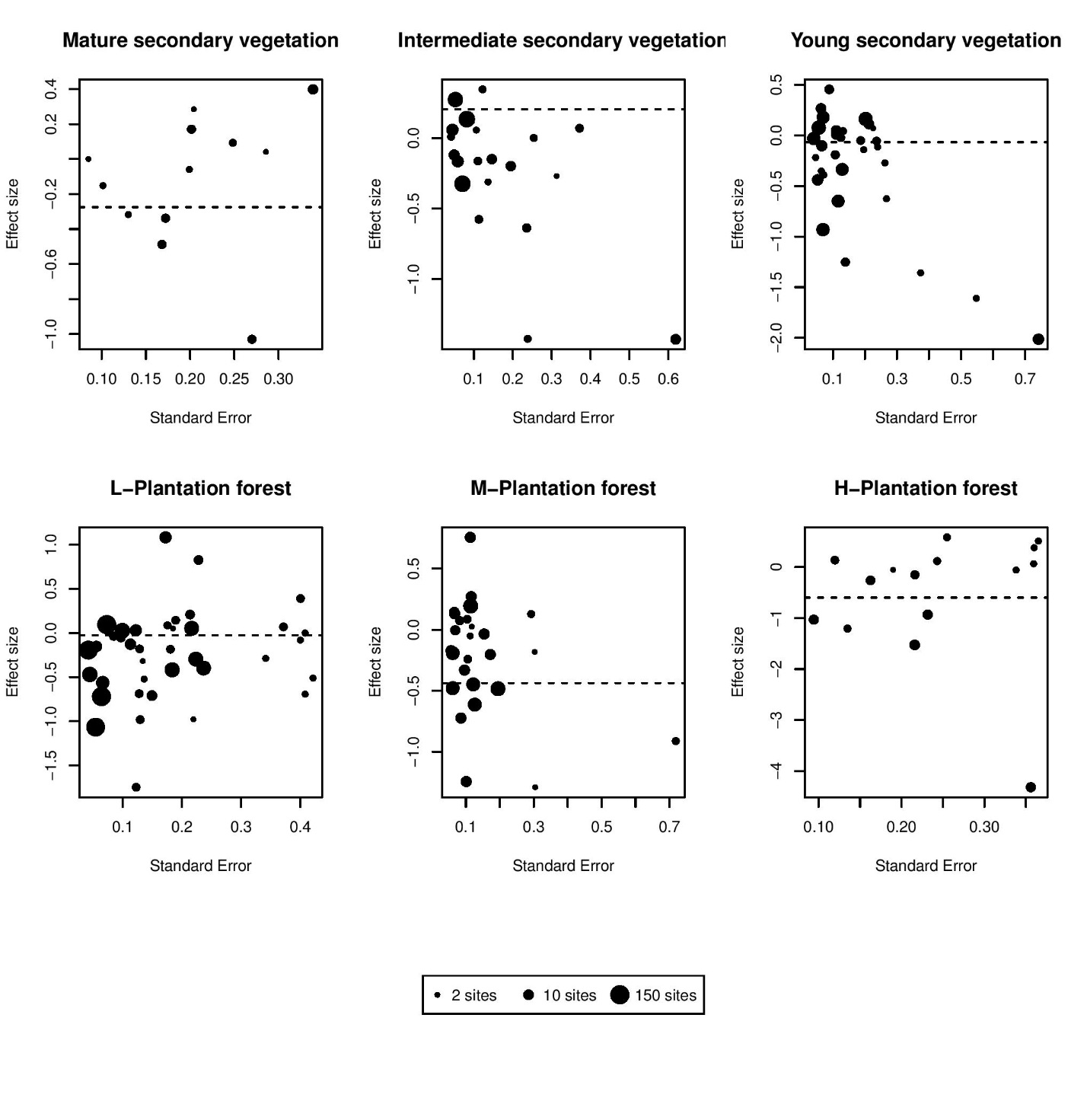
**

**Supplementary Material: Appendix B**

Adum GB, Eichhorn MP, Oduro W, et al (2013) Two-stage recovery of amphibian assemblages following selective logging of tropical forests. Conserv Biol 27:354–363. doi: 10.1111/cobi.12006

Alcala EL, Alcala AC, Dolino CN (2004) Amphibians and reptiles in tropical rainforest fragments on Negros Island, the Philippines. Environ Conserv 31:254–261. doi: 10.1017/s0376892904001407

Arbeláez-Cortés E, Rodríguez-Correa HA, Restrepo-Chica M (2011) Mixed bird flocks: patterns of activity and species composition in a region of the Central Andes of Colombia. Rev Mex Biodivers 82:639–651.

Armbrecht I, Perfecto I, Silverman E (2006) Limitation of nesting resources for ants in Colombian forests and coffee plantations. Ecol Entomol 31:403–410. doi: 10.1111/j.1365-2311.2006.00802.x

Axmacher JC, Brehm G, Hemp A, et al (2009) Determinants of diversity in afrotropical herbivorous insects (Lepidoptera: Geometridae): plant diversity, vegetation structure or abiotic factors? J Biogeogr 36:337–349. doi: 10.1111/j.1365-2699.2008.01997.x

Azhar B, Lindenmayer DB, Wood J, et al (2013) The influence of agricultural system, stand structural complexity and landscape context on foraging birds in oil palm landscapes. Ibis (Lond 1859) 155:297–312. doi: 10.1111/ibi.12025

Bakayoko A, Martin P, Chatelain C, et al (2011) Diversity, family dominance, life forms and ecological strategies of forest fragments compared to continuous forest in southwestern Côte d’Ivoire. Candollea 66:255–262. doi: 10.15553/c2011v662a2

Banks JE, Sandvik P, Keesecker L (2007) Beetle (Coleoptera) and spider (Araneae) diversity in a mosaic of farmland, edge, and tropical forest habitats in western Costa Rica. Pan-Pac Entomol 83:152–160. doi: 10.3956/0031-0603-83.2.152

Barlow J, Gardner TA, Araujo IS, et al (2007a) Quantifying the biodiversity value of tropical primary, secondary, and plantation forests. Proc Natl Acad Sci 104:18555–18560.

Barlow J, Mestre LAM, Gardner TA, Peres CA (2007b) The value of primary, secondary and plantation forests for Amazonian birds. Biol Conserv 136:212–231. doi: 10.1016/j.biocon.2006.11.021

Barlow J, Overal WL, Araujo IS, et al (2007c) The value of primary, secondary and plantation forests for fruit-feeding butterflies in the Brazilian Amazon. J Appl Ecol 44:1001–1012.

Basset Y, Missa O, Alonso A, et al (2008) Changes in arthropod assemblages along a wide gradient of disturbance in Gabon. Conserv Biol 22:1552–1563. doi: 10.1111/j.1523-1739.2008.01017.x

Benedick S, Hill JK, Mustaffa N, et al (2006) Impacts of rain forest fragmentation on butterflies in northern Borneo: species richness, turnover and the value of small fragments. J Appl Ecol 43:967–977. doi: 10.1111/j.1365-2664.2006.01209.x

Berry NJ, Phillips OL, Lewis SL, et al (2010) The high value of logged tropical forests: lessons from northern Borneo. Biodivers Conserv 19:985–997.

Bicknell J, Peres CA (2010) Vertebrate population responses to reduced-impact logging in a neotropical forest. For Ecol Manage 259:2267–2275. doi: 10.1016/j.foreco.2010.02.027

Bobo KS, Waltert M, Fermon H, et al (2006) From forest to farmland: butterfly diversity and habitat associations along a gradient of forest conversion in Southwestern Cameroon. J Insect Conserv 10:29–42. doi: 10.1007/s10841-005-8564-x

Borges SH (2007) Bird assemblages in secondary forests developing after slash-and-burn agriculture in the Brazilian Amazon. J Trop Ecol 23:469–477. doi: 10.1017/s0266467407004105

Bouyer J, Sana Y, Samandoulgou Y, et al (2007) Identification of ecological indicators for monitoring ecosystem health in the trans-boundary W Regional park: a pilot study. Biol Conserv 138:73–88. doi: 10.1016/j.biocon.2007.04.001

Brearley FQ (2011) Below-ground secondary succession in tropical forests of Borneo. J Trop Ecol 27:413–420. doi: 10.1017/s0266467411000149

Cáceres NC, Nápoli RP, Casella J, Hannibal W (2010) Mammals in a fragmented savannah landscape in south-western Brazil. J Nat Hist 44:491–512. doi: 10.1080/00222930903477768

Castro-Luna AA, Sosa VJ, Castillo-Campos G (2007) Bat diversity and abundance associated with the degree of secondary succession in a tropical forest mosaic in south-eastern Mexico. Anim Conserv 10:219–228. doi: 10.1111/j.1469-1795.2007.00097.x

Center for International Forestry Research (CIFOR) (2013a) Multidisciplinary Landscape Assessment - Philippines.

Center for International Forestry Research (CIFOR) (2013b) Multidisciplinary Landscape Assessment - Cameroon.

Centro Agronómico Tropical de Investigación y Enseñanza (CATIE) (2010) Unpublished data of reptilian and amphibian diversity in six countries in Central America.

Cleary DFR, Mooers AO (2006) Burning and logging differentially affect endemic vs. widely distributed butterfly species in Borneo. Divers Distrib 12:409–416. doi: 10.1111/j.1366-9516.2006.00256.x

Cleary DFR, Mooers AO, Eichhorn KAO, et al (2004) Diversity and community composition of butterflies and odonates in an ENSO-induced fire affected habitat mosaic: a case study from East Kalimantan, Indonesia. Oikos 105:426–446. doi: 10.1111/j.0030-1299.2004.12219.x

D’Cruze N, Kumar S (2011) Effects of anthropogenic activities on lizard communities in northern Madagascar. Anim Conserv 14:542–552. doi: 10.1111/j.1469-1795.2011.00459.x

Dallimer M, Parnell M, Bicknell JE, Melo M (2012) The importance of novel and agricultural habitats for the avifauna of an oceanic island. J Nat Conserv 20:191–199. doi: 10.1016/j.jnc.2012.04.001

Danquah E, Oppong SK, Nutsuakor ME (2012) Effect of protected area category on mammal abundance in Western Ghana. J Biodivers Environ Sci 2:50–57.

Davis AL V, Philips TK (2005) Effect of deforestation on a southwest Ghana dung beetle assemblage (Coleoptera: Scarabaeidae) at the periphery of Ankasa conservation area. Environ Entomol 34:1081–1088. doi: 10.1603/0046-225x(2005)034[1081:eodoas]2.0.co;2

de Lima RF, Dallimer M, Atkinson PW, Barlow J (2013) Biodiversity and land-use change: understanding the complex responses of an endemic-rich bird assemblage. Divers Distrib 19:411–422. doi: 10.1111/ddi.12015

Devineau JL, Fournier A, Nignan S (2009) “Ordinary biodiversity” in western Burkina Faso (West Africa): what vegetation do the state forests conserve? Biodivers Conserv 18:2075–2099. doi: 10.1007/s10531-008-9574-2

Dominguez-Haydar Y, Armbrecht I (2010) Response of ants and their seed removal in rehabilitation areas and forests at El Cerrejon coal mine in Colombia. Restor Ecol 19:178–184. doi: 10.1111/j.1526-100x.2010.00735.x

Doulton H, Marsh C, Newman A, et al (2007) Conservation Comores 2005: biodiversity and resource-use assessment and environmental awareness. University of Oxford, U.K.

Endo W, Peres CA, Salas E, et al (2010) Game vertebrate densities in hunted and nonhunted forest sites in Manu National Park, Peru. Biotropica 42:251–261. doi: 10.1111/j.1744-7429.2009.00546.x

Faruk A, Belabut D, Ahmad N, et al (2013) Effects of oil-palm plantations on diversity of tropical anurans. Conserv Biol 27:615–624. doi: 10.1111/cobi.12062

Farwig N, Sajita N, Böhning-Gaese K (2008) Conservation value of forest plantations for bird communities in western Kenya. For Ecol Manage 255:3885–3892. doi: 10.1016/j.foreco.2008.03.042

Fayle TM, Turner EC, Snaddon JL, et al (2010) Oil palm expansion into rain forest greatly reduces ant biodiversity in canopy, epiphytes and leaf-litter. Basic Appl Ecol 11:337–345. doi: 10.1016/j.baae.2009.12.009

Fermon H, Waltert M, Vane-Wright RI, Muhlenberg M (2005) Forest use and vertical stratification in fruit-feeding butterflies of Sulawesi, Indonesia: impacts for conservation. Biodivers Conserv 14:333–350. doi: 10.1007/s10531-004-5054-9

Filgueiras BKC, Iannuzzi L, Leal IR (2011) Habitat fragmentation alters the structure of dung beetle communities in the Atlantic Forest. Biol Conserv 144:362–369.

Floren A, Freking A, Biehl M, Linsenmair KE (2001) Anthropogenic disturbance changes the structure of arboreal tropical ant communities. Ecography 24:547–554. doi: 10.1111/j.1600-0587.2001.tb00489.x

Freire GD, Motta PC (2011) Effects of experimental fire regimes on the abundance and diversity of cursorial arachnids of Brazilian savannah (cerrado biome). J Arachnol 39:263–272. doi: 10.1636/cp10-85.1

Fukuda D, Tisen OB, Momose K, Sakai S (2009) Bat diversity in the vegetation mosaic around a lowland dipterocarp forest of Borneo. Raffles Bull Zool 57:213–221.

García-R JC, Cárdenas-H H, Castro-H F (2007) Relationship between anurans diversity and successional stages of a very humid low montane forest in Valle del Cauca, southwestern of Colombia. Caldasia 29:363–374.

Gardner TA, Hernández MIM, Barlow J, Peres CA (2008) Understanding the biodiversity consequences of habitat change: the value of secondary and plantation forests for neotropical dung beetles. J Appl Ecol 45:883–893.

Gardner TA, Ribeiro-Júnior MA, Barlow J, et al (2007) The value of primary, secondary, and plantation forests for a neotropical herpetofauna. Conserv Biol 21:775–787. doi: 10.1111/j.1523-1739.2007.00659.x

Gomes LGL, Oostra V, Nijman V, et al (2008) Tolerance of frugivorous birds to habitat disturbance in a tropical cloud forest. Biol Conserv 141:860–871. doi: 10.1016/j.biocon.2008.01.007

Gove AD, Majer JD, Rico-Gray V (2005) Methods for conservation outside of formal reserve systems: the case of ants in the seasonally dry tropics of Veracruz, Mexico. Biol Conserv 126:328–338. doi: 10.1016/j.biocon.2005.06.008

Granjon L, Duplantier JM (2011) Guinean biodiversity at the edge: rodents in forest patches of southern Mali. Mamm Biol 76:583–591. doi: 10.1016/j.mambio.2011.06.003

Gray CL, Slade EM, Mann DJ, Lewis OT (2014) Do riparian reserves support dung beetle biodiversity and ecosystem services in oil palm-dominated tropical landscapes? Ecol Evol 4:1049–1060. doi: 10.1002/ece3.1003

Hashim NR, Akmal WF, Jusoh W, Nasir M (2010) Ant diversity in a peninsular Malaysian mangrove forest and oil palm plantation. Asian Myrmecology 3:5–8.

Hawes J, da Silva Motta C, Overal WL, et al (2009) Diversity and composition of Amazonian moths in primary, secondary and plantation forests. J Trop Ecol 25:281–300. doi: 10.1017/s0266467409006038

Henschel P (2008) The Conservation biology of the Leopard *Panthera pardus* in Gabon: status, threats and strategies for conservation. Georg-August-Universität Göttingen

Hietz P (2005) Conservation of vascular epiphyte diversity in Mexican coffee plantations. Conserv Biol 19:391–399. doi: 10.1111/j.1523-1739.2005.00145.x

Higuera D, Wolf JHD (2010) Vascular epiphytes in dry oak forests show resilience to anthropogenic disturbance, Cordillera Oriental, Colombia. Caldasia 32:161–174.

Hilje B, Aide TM (2012) Recovery of amphibian species richness and composition in a chronosequence of secondary forests, northeastern Costa Rica. Biol Conserv 146:170–176. doi: 10.1016/j.biocon.2011.12.007

Horgan FG (2009) Invasion and retreat: shifting assemblages of dung beetles amidst changing agricultural landscapes in central Peru. Biodivers Conserv 18:3519–3541. doi: 10.1007/s10531-009-9658-7

Hylander K, Nemomissa S (2009) Complementary roles of home gardens and exotic tree plantations as alternative habitats for plants of the Ethiopian montane rainforest. Conserv Biol 23:400–409. doi: 10.1111/j.1523-1739.2008.01097.x

Kapoor V (2008) Effects of rainforest fragmentation and shade-coffee plantations on spider communities in the Western Ghats, India. J Insect Conserv 12:53–68. doi: 10.1007/s10841-006-9062-5

Kessler M, Abrahamczyk S, Bos M, et al (2009) Alpha and beta diversity of plants and animals along a tropical land-use gradient. Ecol Appl 19:2142–2156. doi: 10.1890/08-1074.1

Kessler M, Kessler PJA, Gradstein SR, et al (2005) Tree diversity in primary forest and different land use systems in Central Sulawesi, Indonesia. Biodivers Conserv 14:547–560. doi: 10.1007/s10531-004-3914-7

Kone M, Konate S, Yeo K, et al (2010) Diversity and abundance of terrestrial ants along a gradient of land use intensification in a transitional forest-savannah zone of Côte d’Ivoire. J Appl Biosci 29:1809–1827.

Kurz DJ, Nowakowski AJ, Tingley MW, et al (2014) Forest-land use complementarity modifies community structure of a tropical herpetofauna. Biol Conserv 170:246–255. doi: 10.1016/j.biocon.2013.12.027

López-Quintero CA, Straatsma G, Franco-Molano AE, Boekhout T (2012) Macrofungal diversity in Colombian Amazon forests varies with regions and regimes of disturbance. Biodivers Conserv 2221–2243. doi: 10.1007/s10531-012-0280-8

Lachat T, Attignon S, Djego J, et al (2006) Arthropod diversity in Lama forest reserve (South Benin), a mosaic of natural, degraded and plantation forests. Biodivers Conserv 15:3–23. doi: 10.1007/s10531-004-1234-6

Lasky JR, Keitt TH (2010) Abundance of Panamanian dry-forest birds along gradients of forest cover at multiple scales. J Trop Ecol 26:67–78. doi: 10.1017/s0266467409990368

Lehouck V, Spanhove T, Colson L, et al (2009) Habitat disturbance reduces seed dispersal of a forest interior tree in a fragmented African cloud forest. Oikos 118:1023–1034. doi: 10.1111/j.1600-0706.2009.17300.x

Letcher SG, Chazdon RL (2009) Rapid recovery of biomass, species richness, and species composition in a forest chronosequence in northeastern Costa Rica. Biotropica 41:608–617. doi: 10.1111/j.1744-7429.2009.00517.x

Li SN, Zou FS, Zhang Q, Sheldon FH (2013) Species richness and guild composition in rubber plantations compared to secondary forest on Hainan Island, China. Agrofor Syst 87:1117–1128. doi: 10.1007/s10457-013-9624-y

Liow LH, Sodhi NS, Elmqvist T (2001) Bee diversity along a disturbance gradient in tropical lowland forests of south-east Asia. J Appl Ecol 38:180–192. doi: 10.1046/j.1365-2664.2001.00582.x

Lo-Man-Hung NF, Gardner TA, Ribeiro-Júnior MA, et al (2008) The value of primary, secondary, and plantation forests for Neotropical epigeic arachnids. J Arachnol 36:394–401. doi: 10.1636/ct07-136.1

Lo-Man-Hung NF, Marichal R, Candiani DF, et al (2011) Impact of different land management on soil spiders (Arachnida: Araneae) in two Amazonian areas of Brazil and Colombia. J Arachnol 39:296–302. doi: 10.1636/cp10-89.1

MacSwiney MCG, Vilchis PL, Clarke FM, Racey PA (2007) The importance of cenotes in conserving bat assemblages in the Yucatan, Mexico. Biol Conserv 136:499–509. doi: 10.1016/j.biocon.2006.12.021

Malonza PK, Veith M (2012) Amphibian community along elevational and habitat disturbance gradients in the Taita Hills, Kenya. Herpetotropicos 7:7–16.

Marin-Spiotta E, Ostertag R, Silver WL (2007) Long-term patterns in tropical reforestation: plant community composition and aboveground biomass accumulation. Ecol Appl 17:828–839. doi: 10.1890/06-1268

Marsh CJ, Lewis OT, Said I, Ewers RM (2010) Community-level diversity modelling of birds and butterflies on Anjouan, Comoro Islands. Biol Conserv 143:1364–1374. doi: 10.1016/j.biocon.2010.03.010

Massawe AW, Makundi RH, Mulungu LS, et al (2012) Breeding dynamics of rodent species inhabiting farm-fallow mosaic fields in Central Tanzania. African Zool 47:128–137. doi: 10.3377/004.047.0117

Matsumoto T, Itioka T, Yamane S, Momose K (2009) Traditional land use associated with swidden agriculture changes encounter rates of the top predator, the army ant, in Southeast Asian tropical rain forests. Biodivers Conserv 18:3139–3151. doi: 10.1007/s10531-009-9632-4

Mayfield MM, Ackerly D, Daily GC (2006) The diversity and conservation of plant reproductive and dispersal functional traits in human-dominated tropical landscapes. J Ecol 94:522–536. doi: 10.1111/j.1365-2745.2006.01108.x

McNamara S, Erskine PD, Lamb D, et al (2012) Primary tree species diversity in secondary fallow forests of Laos. For Ecol Manage 281:93–99. doi: 10.1016/j.foreco.2012.06.004

Milder JC, DeClerck FAJ, Sanfiorenzo A, et al (2010) Effects of farm and landscape management on bird and butterfly conservation in western Honduras. Ecosphere 1:art2. doi: 10.1890/es10-00003.1

Muchane MN, Karanja D, Wambugu GM, et al (2012) Land use practices and their implications on soil macro-fauna in Maasai Mara ecosystem. Int J Biodivers Conserv 4:500–514. doi: 10.5897/ijbc12.030

Munyekenye FB, Mwangi EM, Gichuki NN (2008) Bird species richness and abundance in different forest types at Kakamega Forest, western Kenya. Ostrich 79:37–42. doi: 10.2989/ostrich.2008.79.1.4.361

Naidoo R (2004) Species richness and community composition of songbirds in a tropical forest-agricultural landscape. Anim Conserv 7:93–105. doi: 10.1017/s1367943003001185

Nakagawa M, Miguchi H, Nakashizuka T (2006) The effects of various forest uses on small mammal communities in Sarawak, Malaysia. For Ecol Manage 231:55–62. doi: 10.1016/j.foreco.2006.05.006

Navarrete D, Halffter G (2008) Dung beetle (Coleoptera: Scarabaeidae: Scarabaeinae) diversity in continuous forest, forest fragments and cattle pastures in a landscape of Chiapas, Mexico: the effects of anthropogenic changes. Biodivers Conserv 17:2869–2898. doi: 10.1007/s10531-008-9402-8

Ngai JT, Kirby KR, Gilbert B, et al (2008) The impact of land-use change on larval insect communities: testing the role of habitat elements in conservation. Ecoscience 15:160–168. doi: 10.2980/15-2-3098

Nicolas V, Barriere P, Tapiero A, Colyn M (2009) Shrew species diversity and abundance in Ziama Biosphere Reserve, Guinea: comparison among primary forest, degraded forest and restoration plots. Biodivers Conserv 18:2043–2061. doi: 10.1007/s10531-008-9572-4

Noriega JA, Palacio JM, Monroy-G JD, Valencia E (2012) Estructura de un ensamblaje de escarabajos coprofagos (Coleoptera: Scarabaeinae) en tres sitios con diferente uso del suelo en Antioquia, Colombia. Actual Biol 34:43–54.

Noriega JA, Realpe E, Fagua G (2007) Diversidad de escarabajos coprofagos (Coleoptera: Scarabaeidae) en un bosque de galeria con tres estadios de alteracion. Univ Sci 12:51–63.

Nöske NM, Hilt N, Werner FA, et al (2008) Disturbance effects on diversity of epiphytes and moths in a montane forest in Ecuador. Basic Appl Ecol 9:4–12. doi: 10.1016/j.baae.2007.06.014

Nyeko P (2009) Dung beetle assemblages and seasonality in primary forest and forest fragments on agricultural landscapes in Budongo, Uganda. Biotropica 41:476–484. doi: 10.1111/j.1744-7429.2009.00499.x

O’Dea N, Whittaker RJ (2007) How resilient are Andean montane forest bird communities to habitat degradation? Biodivers Conserv 16:1131–1159. doi: 10.1007/s10531-006-9095-9

Owiunji I, Plumptre AJ (1998) Bird communities in logged and unlogged compartments in Budongo Forest, Uganda. For Ecol Manage 108:115–126. doi: 10.1016/s0378-1127(98)00219-9

Parry L, Barlow J, Peres CA (2009) Hunting for sustainability in tropical secondary forests. Conserv Biol 23:1270–1280. doi: 10.1111/j.1523-1739.2009.01224.x

Phalan B, Onial M, Balmford A, Green RE (2011) Reconciling food production and biodiversity conservation: land sharing and land sparing compared. Science 333:1289–1291.

Presley SJ, Willig MR, Wunderle Joseph M. J, Saldanha LN (2008) Effects of reduced-impact logging and forest physiognomy on bat populations of lowland Amazonian forest. J Appl Ecol 45:14–25. doi: 10.1111/j.1365-2664.2007.01373.x

Ranganathan J, Daniels RJR, Chandran MDS, et al (2008) Sustaining biodiversity in ancient tropical countryside. Proc Natl Acad Sci U S A 105:17852–17854.

Reid JL, Harris JBC, Zahawi RA (2012) Avian habitat preference in tropical forest restoration in southern Costa Rica. Biotropica 44:350–359. doi: 10.1111/j.1744-7429.2011.00814.x

Rey-Velasco JC, Miranda-Esquivel DR (2012) Unpublished data of the response of ground beetles (Coleoptera: Carabidae) in the northeastern Colombian Andes to habitat modification.

Richardson BA, Richardson MJ, Soto-Adames FN (2005) Separating the effects of forest type and elevation on the diversity of litter invertebrate communities in a humid tropical forest in Puerto Rico. J Anim Ecol 74:926–936. doi: 10.1111/j.1365-2656.2005.00990.x

Romero-Duque LP, Jaramillo VJ, Perez-Jimenez A (2007) Structure and diversity of secondary tropical dry forests in Mexico, differing in their prior land-use history. For Ecol Manage 253:38–47. doi: 10.1016/j.foreco.2007.07.002

Rosselli L (2011) Factores ambientales relacionados con la presencia y abundancia de las aves de los humedales de la Sabana de Bogotá. Universidad Nacional de Colombia

Roth DS, Perfecto I, Rathcke B (1994) The effects of management systems on ground-foraging ant diversity in Costa Rica. Ecol Appl 4:423–436. doi: 10.2307/1941947

Rousseau L, Fonte SJ, Tellez O, et al (2013) Soil macrofauna as indicators of soil quality and land use impacts in smallholder agroecosystems of western Nicaragua. Ecol Indic 27:71–82. doi: 10.1016/j.ecolind.2012.11.020

Safian S, Csontos G, Winkler D (2011) Butterfly community recovery in degraded rainforest habitats in the Upper Guinean Forest Zone (Kakum forest, Ghana). J Insect Conserv 15:351–359. doi: 10.1007/s10841-010-9343-x

Sakchoowong W, Nomura S, Ogata K, Chanpaisaeng J (2008) Diversity of pselaphine beetles (Coleoptera: Staphylinidae: Pselaphinae) in eastern Thailand. Entomol Sci 11:301–313. doi: 10.1111/j.1479-8298.2008.00281.x

Schmitt CB, Senbeta F, Denich M, et al (2010) Wild coffee management and plant diversity in the montane rainforest of southwestern Ethiopia. Afr J Ecol 48:78–86. doi: 10.1111/j.1365-2028.2009.01084.x

Sedlock JL, Weyandt SE, Cororan L, et al (2008) Bat diversity in tropical forest and agro-pastoral habitats within a protected area in the Philippines. Acta Chiropterologica 10:349–358. doi: 10.3161/150811008x414926

Shafie NJ, Sah SAM, Latip NSA, et al (2011) Diversity pattern of bats at two contrasting habitat types along Kerian River, Perak, Malaysia. Trop Life Sci Res 22:13–22.

Sheil D, Puri RK, Basuki I, et al (2002) Exploring biological diversity, environment and local people’s perspectives in forest landscapes: methods for a multidisciplinary landscape assessment. Jakarta, Indonesia

Sheldon FH, Styring A, Hosner PA (2010) Bird species richness in a Bornean exotic tree plantation: a long-term perspective. Biol Conserv 143:399–407. doi: 10.1016/j.biocon.2009.11.004

Slade EM, Mann DJ, Lewis OT (2011) Biodiversity and ecosystem function of tropical forest dung beetles under contrasting logging regimes. Biol Conserv 144:166–174. doi: 10.1016/j.biocon.2010.08.011

Smith-Pardo A, Gonzalez VH (2007) Diversidad de abejas (Hymenoptera: Apoidea) en estados sucesionales del bosque humedo tropical. Acta Biológica Colomb 12:43–55.

Sodhi NS, Wilcove DS, Lee TM, et al (2010) Deforestation and avian extinction on tropical landbridge islands. Conserv Biol 24:1290–1298. doi: 10.1111/j.1523-1739.2010.01495.x

Soh MCK, Sodhi NS, Lim SLH (2006) High sensitivity of montane bird communities to habitat disturbance in Peninsular Malaysia. Biol Conserv 129:149–166. doi: 10.1016/j.biocon.2005.10.030

Sridhar H, Raman TRS, Mudappa D (2008) Mammal persistence and abundance in tropical rainforest remnants in the southern Western Ghats, India. Curr Sci 94:748–757.

Stouffer PC, Johnson EI, Bierregaard Richard O. J, Lovejoy TE (2011) Understory bird communities in Amazonian rainforest fragments: species turnover through 25 years post-isolation in recovering landscapes. PLoS One. doi: 10.1371/journal.pone.0020543

Struebig MJ, Kingston T, Zubaid A, et al (2008) Conservation value of forest fragments to Palaeotropical bats. Biol Conserv 141:2112–2126. doi: 10.1016/j.biocon.2008.06.009

Sung YH, Karraker NE, Hau BCH (2012) Terrestrial herpetofaunal assemblages in secondary forests and exotic *Lophostemon confertus* plantations in South China. For Ecol Manage 270:71–77. doi: 10.1016/j.foreco.2012.01.011

Sutrisno H (2010) The impact of human activities to dynamic of insect communities: a case study in Gunung Salak, West Java. HAYATI J Biosci 17:161–166. doi: 10.4308/hjb.17.4.161

Tonhasca Jr. A, Blackmer JL, Albuquerque GS (2002) Abundance and diversity of Euglossine bees in the fragmented landscape of the Brazilian Atlantic Forest. Biotropica 34:416–422. doi: 10.1646/0006-3606(2002)034

Turner EC, Foster WA (2009) The impact of forest conversion to oil palm on arthropod abundance and biomass in Sabah, Malaysia. J Trop Ecol 25:23–30. doi: 10.1017/s0266467408005658

Tylianakis JM, Klein A-M, Tscharntke T (2005) Spatiotemporal variation in the diversity of Hymenoptera across a tropical habitat gradient. Ecology 86:3296–3302.

Urbina-Cardona JN, Londoño-Murcia MC, García-Ávila DG (2008) Spatio-temporal dymanics of snake diversity in four habitats with different degrees of anthropogenic disturbance in the Gorgona Island National Natural Park in the Colombian Pacific. Caldasia 30:479–493.

Urbina-Cardona JN, Olivares-Perez M, Reynoso VH (2006) Herpetofauna diversity and microenvironment correlates across a pasture-edge-interior ecotone in tropical rainforest fragments in the Los Tuxtlas Biosphere Reserve of Veracruz, Mexico. Biol Conserv 132:61–75. doi: 10.1016/j.biocon.2006.03.014

Vasconcelos HL (1999) Effects of forest disturbance on the structure of ground-foraging ant communities in central Amazonia. Biodivers Conserv 8:409–420.

Vasconcelos HL, Pacheco R, Silva RC, et al (2009) Dynamics of the leaf-litter arthropod fauna following fire in a neotropical woodland savanna. PLoS One 4:9. doi: 10.1371/journal.pone.0007762

Vasconcelos HL, Vilhena JMS, Caliri GJA (2000) Responses of ants to selective logging of a central Amazonian forest. J Appl Ecol 37:508–514. doi: 10.1046/j.1365-2664.2000.00512.x

Vergara CH, Badano EI (2009) Pollinator diversity increases fruit production in Mexican coffee plantations: the importance of rustic management systems. Agric Ecosyst Environ 129:117–123. doi: 10.1016/j.agee.2008.08.001

Virgilio M, Backeljau T, Emeleme R, et al (2011) A quantitative comparison of frugivorous tephritids (Diptera: Tephritidae) in tropical forests and rural areas of the Democratic Republic of Congo. Bull Entomol Res 101:591–597. doi: 10.1017/s0007485311000216

Vu LV (2009) Diversity and similarity of butterfly communities in five different habitat types at Tam Dao National Park, Vietnam. J Zool 277:15–22. doi: 10.1111/j.1469-7998.2008.00498.x

Vu LV (2005) Unpublished data of diversity and similarity of butterfly communities in five different habitat types at Tam Dao National Park, Vietnam.

Vu LV, Quang VC (2011) Diversity Pattern of Butterfly Communities (Lepidoptera, Papilionoidae) in Different Habitat Types in a Tropical Rain Forest of Southern Vietnam. ISRN Zool 2011:1–8. doi: 10.5402/2011/818545

Watling JI, Gerow K, Donnelly MA (2009) Nested species subsets of amphibians and reptiles on Neotropical forest islands. Anim Conserv 12:467–476. doi: 10.1111/j.1469-1795.2009.00274.x

Wells K, Kalko EK V, Lakim MB, Pfeiffer M (2007) Effects of rain forest logging on species richness and assemblage composition of small mammals in Southeast Asia. J Biogeogr 34:1087–1099. doi: 10.1111/j.1365-2699.2006.01677.x

Wiafe ED, Amfo-Otu R (2012) Forest duiker (*Cephalophus* spp.) abundance and hunting activities in the Kakum conservation area, Ghana. J Ecol Nat Environ 4:114–118. doi: 10.5897/jene11.144

Willig MR, Presley SJ, Bloch CP, et al (2007) Phyllostomid bats of lowland Amazonia: effects of habitat alteration on abundance. Biotropica 39:737–746. doi: 10.1111/j.1744-7429.2007.00322.x

Wronski T, Gilbert K, Long E, et al (2014) Species richness and meta-community structure of land snails along an altitudinal gradient on Bioko Island, Equatorial Guinea. J Molluscan Stud 80:161–168. doi: 10.1093/mollus/eyu008

Wunderle JM, Henriques LMP, Willig MR (2006) Short-term responses of birds to forest gaps and understory: an assessment of reduced-impact logging in a lowland Amazon forest. Biotropica 38:235–255. doi: 10.1111/j.1744-7429.2006.00138.x

**Supplementary Material: Figure S2**

**Supplementary Figure S2:** Box plot showing, at each site within each land use, the proportion of species with GBIF records, used for estimating species' range size. Results are split by continent (Asia, Africa, Central America and South America). Primary = Primary vegetation, MSV = Mature secondary vegetation, ISV = Intermediate secondary vegetation, YSV = Young secondary vegetation, L-Plantation = low-intensity plantation forest, M-Plantation = medium-intensity plantation forest, and H-Plantation = high-intensity plantation forest. A large number of sites in Africa and South America are considered outliers and have low proportion of GBIF records, which could explain the small variation in CWM log_e_ range size results.

**
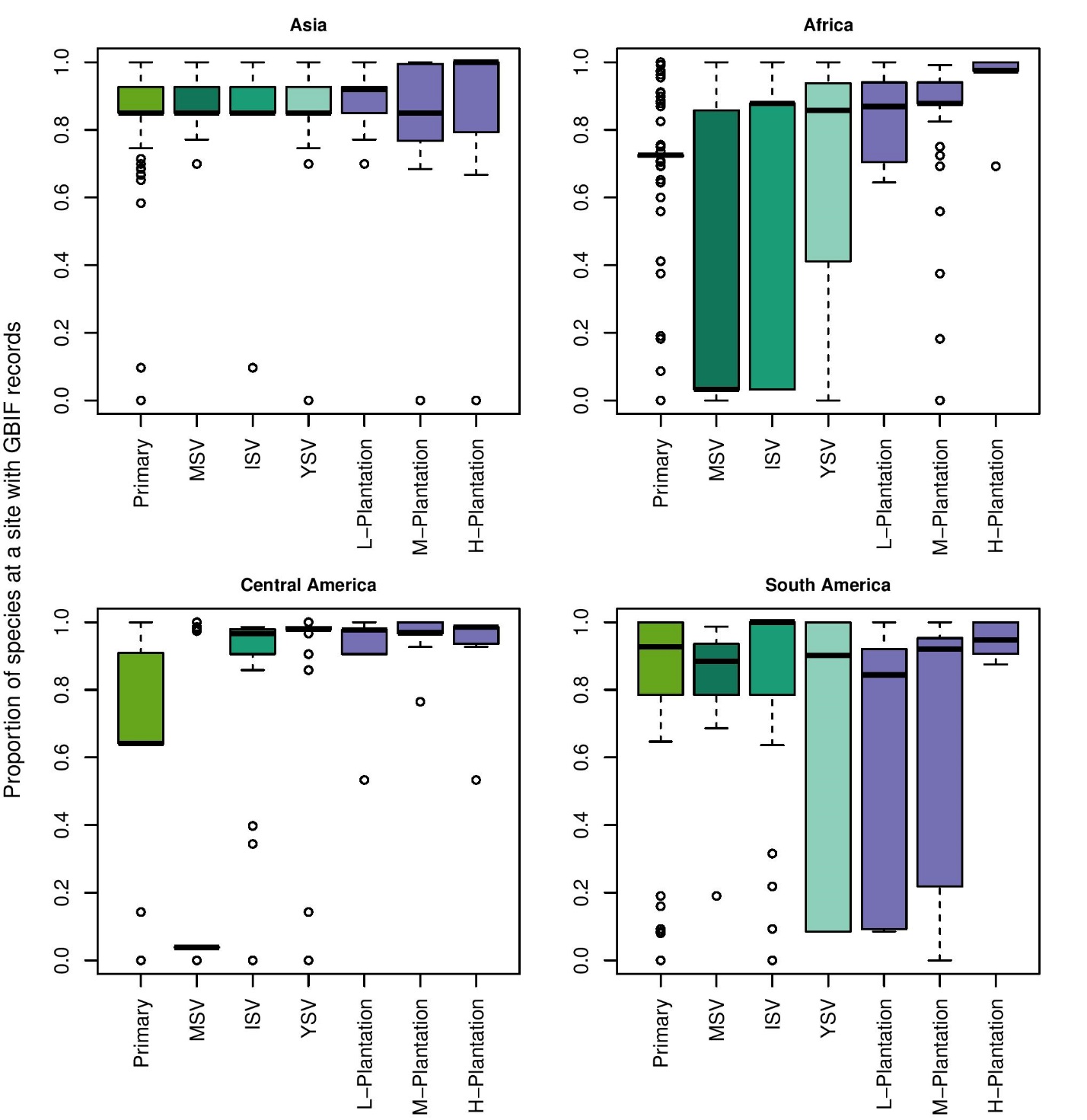
**

**Supplementary Table S1**

**Supplementary Table S1:** Distribution of sites within each continent in each of the tropical biomes, as well as the total number of sites in each biome.

| Biome | Asia | Africa | Central America | South America | All |
| --- | --- | --- | --- | --- | --- |
| Montane Grasslands & Shrublands | 22 | 233 | 0 | 0 | 255 |
| Deserts & Xeric Shrublands | 0 | 0 | 0 | 13 | 13 |
| Tropical & Subtropical Grasslands, Savannas & Shrublands | 0 | 656 | 0 | 337 | 993 |
| Tropical & Subtropical Coniferous Forests | 0 | 0 | 128 | 0 | 128 |
| Tropical & Subtropical Dry Broadleaf Forests | 3 | 9 | 56 | 24 | 92 |
| Tropical & Subtropical Moist Broadleaf Forests | 1014 | 1292 | 539 | 568 | 3413 |
| Mangroves | 0 | 0 | 7 | 0 | 7 |
